# Effect Modification by Environmental Quality on the Association between Heatwaves and Mortality in Alabama, United States

**DOI:** 10.3390/ijerph14101143

**Published:** 2017-09-28

**Authors:** Yun Jian, Connor Y. H. Wu, Julia M. Gohlke

**Affiliations:** 1 Informatics Institute, University of Alabama at Birmingham, Birmingham, AL 35294, USA; 2 Department of Population Health Sciences, Virginia Polytechnic Institute and State University, Blacksburg, VA 24061, USA; yuhaowu@vt.edu (C.Y.H.W.); jgohlke@vt.edu (J.M.G.)

**Keywords:** heatwaves, non-accidental deaths, cumulative environmental quality, metropolitan counties, Alabama

## Abstract

*Background*: Previous studies have shown that heatwaves are associated with increased mortality. However, it remains unclear whether the associations between heatwaves and mortality are modified by the environmental quality. *Methods*: We used the United States (US) Environmental Protection Agency’s Environmental Quality Index (EQI) and its five domain indices (air, water, land, built, and sociodemographic) to represent the cumulative environmental quality. We applied a time-stratified case-crossover design to analyze the disparities in the association between heatwaves and non-accidental deaths (NAD) among counties with different environmental qualities, in metropolitan areas in Alabama (AL), United States. *Results*: We found significant associations between heatwaves and NAD and a significant effect modification of this relationship by EQI. There were higher odds ratios in counties with the worst cumulative environmental qualities compared to counties with the best cumulative environmental qualities. For example, the percent change in odds ratio (mean and (95% CI)) between heatwave days and non-heatwave days was −10.3% (−26.6, 9.6) in counties with an overall EQI of 1 (the best overall environment) and 13.2% (4.9, 22.2) in counties with an overall EQI of 3 (the worst overall environment). Among the five domains, air quality had the strongest effect modification on the association. *Conclusion*: Our findings provide evidence that the associations between heatwaves and NAD vary among areas with different environmental qualities. These findings suggest that integration of air quality and heatwave warning systems may provide greater protection to public health.

## 1. Introduction

Numerous previous studies have reported increased mortality during heatwaves worldwide [1,2,3,4,5,6,7]. However, the associations varied greatly across different locations. For example, the estimated increase in mortality was about 3% at the national level for the United States (US), while regional increases in mortality on heatwave days compared to non-heatwave days, ranged from 1.84% to 6.76% [7]. Another study found that the increase in mortality in Europe during heat wave days ranged from 7.6% to 33.6% [8].

Previously, both individual and area-level factors have been used to explain variations in the associations between heatwaves and mortality. At the individual level, factors such as age, sex, race, personal education, employment, and medical conditions have been reported to modify temperature-related mortality [9,10]. For example, Schwartz (2005) found that among medical conditions, patients with diabetes had a higher risk of dying on hot days than other subjects, and among sociodemographic characteristics, nonwhites had a greater risk [9]. Yang et al. (2012) reported significant effect modifications by personal education and employment on the association between daily temperature and mortality in Guangzhou China [10]. At the area level, factors such as regional socioeconomic status, climate, and rurality have been used to explain variations in associations between heatwaves and mortality [7,11,12]. For example, Rey et al. (2009) found that excess mortality rates due to heat were twofold higher in the most deprived areas compared to the least deprived areas, in Paris, France [12]. Anderson and Bell (2011) found that heatwave mortality impacts were more pronounced in the Northeast and Midwest compared with the South, among 43 US cities [7]. Medina-Ramón and Schwartz (2007) found that heat effects on mortality were largest in cities with milder summers, less air conditioning and higher population density, among 50 US cities [11]. While those factors are important and helpful for identifying populations that are the most vulnerable to heatwaves, it still remains unclear whether, and how, associations between heatwaves and mortality vary in populations exposed to different environmental qualities.

The environmental quality can influence heatwave-related mortality in multiple ways. For example, one study found that urban water bodies and urban vegetation can mitigate the association between heatwaves and mortality in populations >65 years old by reducing local temperatures [13]. Another study found that populations living in areas with higher ozone concentrations had stronger associations between temperature and cardiovascular mortality [14]. Several studies have adjusted for individual air pollutant concentrations (such as particulate matter and ozone) in models examining the relationship between temperature and mortality [6,8,11,15].

Although the environmental quality may influence associations between heatwaves and health outcomes, formal assessments of the disparities in the associations among areas with different environmental qualities are rare [14,15]. Basu (2009) reviewed literature examining the relationship between high ambient temperature and mortality in 2001–2008, with a focus on how air pollutants may influence the relationship [16]. They found that the effect modification or confounding on high temperature-related mortality varied by the type of air pollutants, and by study location and design. Investigations into the potential modification by other aspects of environmental quality (such as ambient water, built, and land environment) on the health effects of heatwaves are still lacking.

Additionally, the exposure to multiple environmental factors often occurs simultaneously—a population can be exposed to potentially positive (e.g., greenspace) and harmful (e.g., air pollutants) environmental factors at the same time. This requires us to consider the cumulative environmental quality from different domains (aspects of the environment such as air, water, and the built environment) when studying variations in associations between heatwaves and health outcomes under different environments. However, most of the existing studies focus on a single or a few environmental factors in the same domain, such as pollutants in the air domain [8,11,14], possibly due to the challenge of quantifying the overall cumulative environmental quality.

Hence, in this study, we aimed to analyze how the association between heatwave and non-accidental deaths (NAD) may change in counties with different cumulative environmental qualities (effect modification) in metropolitan areas in the State of Alabama (AL), United States (US). We used the Environmental Quality Index (EQI), previously developed by the US Environmental Protection Agency to represent the cumulative environmental quality, on a county-level basis [17,18,19]. We used individual-level mortality data (1997–2010) and multiple heatwave indices to analyze (1) how associations between heatwaves and non-accidental deaths vary by the overall cumulative environmental quality and (2) how the associations vary by different domains of environmental quality.

## 2. Materials and Methods

### 2.1. Mortality Data

We obtained records of deaths that occurred between May and September in the years 1997–2010 from the Alabama Department of Public Health (ADPH; Montgomery, AL, USA). Death records indicated the date of death, the deceased’s residential address, and the cause of death, coded by the International Classification of Diseases 9th (ICD-9) or 10th (ICD-10) revision [20,21]. Details of the data have been described previously [4]. The residential address was geocoded using the same methods in Porter et al. [22] and matched to heatwave index data described below. Briefly, latitude and longitude coordinates of current residences were determined through two rounds of geocoding, based on street address and city data, using ArcGIS 10.0 (ESRI, Redlands, CA, USA). Deaths records that were not able to be geocoded were excluded (3982 death records, 2.8%). In this analysis, we focused on 140,231 non-accidental deaths (ICD-9 codes < 800, and ICD-10 codes A–R) and 58 heat-related deaths (ICD-9 E900 (death due to excessive heat), and ICD-10 X30 (exposure to excessive natural heat)), in 1997–2010, in metropolitan counties of Alabama. The heat-related deaths were combined with non-accidental deaths in the analyses, due to their small number, and the combined death records are referred to as NAD hereafter. Although accidental deaths may also be influenced by heatwaves, the relationship between accidental deaths and heatwaves and how the relationship can be modified by the environmental quality can differ from that of NAD [23]. Thus, we excluded accidental deaths in our analyses, as in previous studies [4]. The analysis was limited to metropolitan counties, since a limited number of deaths during heatwaves occurred in non-metropolitan counties. This study was approved by Virginia Tech Institutional Review Board (Protocol #15-1145).

### 2.2. Heatwave Indices

We used three heatwave indices (HI), previously defined in Smith et al. (2013), in our study [24]. Briefly, temperature data on a 12.5 km grid for 1997–2010, obtained from Phase 2 of the North American Land Data Assimilation System (NLDAS-2), were used to develop daily HI [24]. NLDAS-2 provides the air temperature at 2 m above the surface, which is the product of data assimilation systems using weather observation data from weather stations and also satellite-derived datasets. In this study, we focused on the relative heatwave indices (HI01, HI02, and HI07) in Smith et al. (2013), because a previous study in Alabama suggested stronger associations with adverse health outcomes (death and preterm birth) using these definitions [4]. Specifically, HI01and H02 defined a heat wave as the attainment of a threshold based on the long-term local temperature record for at least two consecutive days. HI01 used 1.645 × standard deviation + mean of daily mean temperature (95th percentile of a normal distribution), and HI02 used 1.282 × standard deviation + mean of daily mean temperature, as the threshold (90th percentile of a normal distribution). In HI07, a heat wave was defined by two thresholds: the 97.5th percentile (T1) and 81st percentile (T2) of daily maximum temperatures. A period was defined as heatwave days for HI07 (1) if there were at least 3 days in the period with a daily maximum temperature above T1, (2) the daily maximum temperature was above T2 for every day of the period, and (3) the average of daily maximum temperature over the entire period was above T1 [24,25]. The thresholds were based on the period between May–September of 1990 to 2011 by a previous study [4]. The number of heatwave days per year using each of the indices was approximately 1.3 using HI01, 5 days per year using HI02, and 1.8 days per year using HI07.

### 2.3. Environmental Quality Index (EQI)

We used the EQI to represent the cumulative environmental quality and study effect modification for the association between heatwaves and NAD. The EQI was constructed to describe the ambient county-level environmental conditions to which residents are exposed [17,18] and was designed to represent simultaneous exposure to several different potential sources of environmental factors. Previously, the EQI has been applied to estimate associations between the cumulative environmental quality and preterm birth, cancer incidence, and non-accidental mortality in the U.S. [19,26,27].

Development of the EQI included two levels of Principal Component Analysis (PCA) to reduce 219 environmental variables into a set of county level indices in the U.S. for 2000–2005 [18,28]. In the first PCA, individual environmental variables from five aspects of environmental quality were obtained and categorized into domain-specific indices (air, water, land, built and sociodemographic). In the second PCA, these five indices were used to generate an overall environmental quality index. In both of the PCA steps, only the first principal component was retained. Based on the loading (weights) of each environmental variable in the PCA, higher values on the EQI represent a worse environmental quality. The air EQI includes 87 variables covering both criteria air pollutants and hazardous air pollutants. The water EQI includes 80 variables, covering overall water quality, general water contamination, recreational water quality, domestic use, drought, and chemical contamination. The land EQI includes 26 variables, covering agriculture, contaminants, facilities, and radon. The built EQI includes 14 variables, covering roads, public transit, business environments, and subsidized housing environments. Finally, the sociodemographic EQI includes 12 variables for socioeconomic environment and crime. The overall and domain-specific EQI data were downloaded from the US Environment Protection Agency (EPA) [29].

EQI is available both as unstratified and stratified indices. For the unstratified indices, all the counties were pooled together in the PCA processes, resulting in the same set of loadings for different environmental variables across rural-urban status. For the stratified indices, PCA was performed separately for counties in each urban-rural stratum, resulting in four different sets of loadings for environmental variables across the urban-rural gradient [19,20]. The urban-rural classification was represented by the Rural Urban Continuum Codes (RUCC) [30]. The original nine RUCC groups were condensed into four groups, as has been done previously: metropolitan urbanized (RUCC1), non-metropolitan urbanized (RUCC2), less urbanized (RUCC3), and thinly populated (RUCC4) areas [31,32]. Due to the small number of counties and limited number of deaths during heatwaves in each EQI tertile in RUCC 2–4 in AL, and potential heterogeneity in environmental qualities across urbanicity, we focused on the stratified EQI in RUCC1 (metropolitan areas) in our analysis.

### 2.4. Statistical Analyses

**Figure 1:**
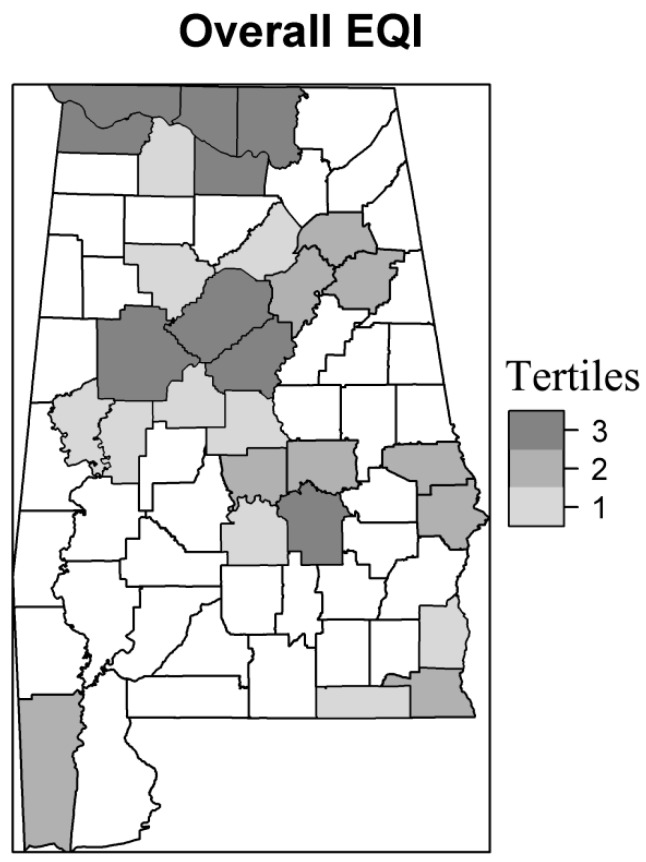
Tertiles for overall Environmental Quality Index (EQI) in metropolitan counties in Alabama. Non-metropolitan counties are white. Tertile 1: counties with the best overall environmental quality in metropolitan areas; tertile 2: counties with median level overall quality; tertile 3: counties with the worst overall environmental quality.

We used a time-stratified case-crossover design to model the associations between heatwaves and mortality in counties with different environmental qualities, which accounts for individual-level differences in sociodemographic variables by using cases as their own control [4,33,34,35,36]. We used conditional logistic regression to estimate the odds ratios (OR) for associations between heatwaves and mortality. Control periods were selected as all days which were on the same day of the week and within the same month as the case day. The results were reported as percent differences in the odds in NAD between heatwave days and non-heatwave days (percent difference = (OR − 1) × 100). To assess for effect modification in the associations between heatwaves and NAD by environmental quality, we included interaction terms between the EQI (overall EQI and the five domain indices) and heatwave indices in the models. Stratified models were also built for the overall EQI and the five EQI indices separately. We grouped counties based on EQI tertiles (Figure 1 and Supplementary Materials). The associations between heatwaves and NAD in counties with EQI 2–3 were compared with the counties with EQI 1 (the best environmental quality).

## 3. Results

Among the 67 counties in Alabama, 28 were classified as metropolitan areas (RUCC 1). Table 1 shows the number of metropolitan counties and non-accidental deaths (NAD) in each EQI tertile. For example, 10 counties were classified in the overall EQI 1 (the best cumulative environmental quality) and 13,805 deaths occurred in these counties between May–September in 1997–2010. Among these deaths, 155 occurred during a heatwave, defined using HI01, 651 happened during HI02 and 243 occurred during HI07.

Interaction models showed that the associations between HIs and NAD differed significantly in counties with different cumulative environmental qualities (represented by the overall EQI). Larger associations were observed in counties with the worst cumulative environmental quality (overall EQI tertile 3) compared to counties with the best cumulative environmental quality (overall EQI tertile 1) for all the three indices studied (*p* values ≤ 0.05, Table S1).

Among the five environmental domains, a significant positive modification was observed when the association in counties with an air tertile of 3 (worst air quality) was compared to that for counties with an air tertile of 1 (best air quality) for all the three heatwave indices (*p* values ≤ 0.05, Table S1). Interaction terms were insignificant for the other EQI domain indices. However, we also observed that most of the interaction terms were positive when the results for the EQI domain indices, tertiles 2 and 3, were compared to the results in tertile 1 (Table S1). The increasing trends in the associations between NAD and heatwaves from the counties with the best (tertile 1) to worst environmental qualities (tertile 3) suggested that harmful health effects of heatwaves were more likely to be seen in areas with worse environmental qualities.

**Figure 2:**
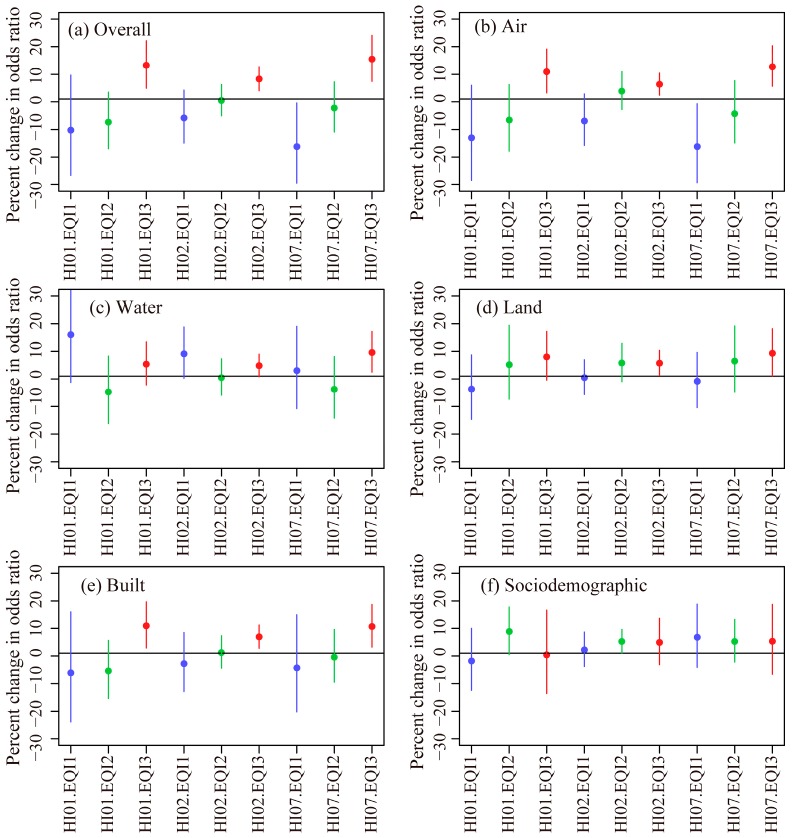
Percent change in odds ratio comparing heatwave days (HI01, HI02, and HI07) and non-heatwave days by tertiles of overall EQI and EQI domain indices, in metropolitan counties in Alabama. A higher EQI tertile represents a worse environmental quality.

Stratified models showed that the percent changes in the odds ratio, comparing heatwave days to non-heatwave days, were mostly positive or insignificant for all metropolitan counties in Alabama (Table 2 and Figure 2). These positive percent changes suggested higher risks for NAD during heatwave days compared to non-heatwave days. Models for the overall EQI resulted in significant positive associations between heatwaves and NAD in counties with an overall EQI tertile of 3 (the worst cumulative environmental quality) for all three heatwave indices, and significant negative associations in counties with overall an EQI tertile of 1 for HI07.

Among the five domain indices, the models for the air EQI showed significant positive percent changes in odds for all three HIs in counties with air EQI tertiles of 3, and significant negative percent changes for HI07 in counties with an air EQI tertile of 1 (Table 2). The models for the water EQI showed significant positive percent changes for HI02 in counties with tertiles of 1 and 3, and for HI07 in counties with tertiles of 3. The model for the land EQI showed significant positive percent changes for HI02 and HI07 in tertile 3. The model for the built EQI showed significant positive percent changes for all HI indices in tertile 3. Finally, the model for the sociodemographic EQI showed significant positive percent changes for HI01 and HI02 in the 2nd tertile (mid-range) counties for sociodemographic EQI. Other percent changes in odds ratios were insignificant.

## 4. Discussion

In this study, we examined the effect modifications of associations between heatwave indices and NAD by overall environmental quality and by different domains of environmental quality. We observed significant variations in associations among counties with different environmental qualities–an increasing trend for associations between NAD and heatwaves from counties with good to poor environmental qualities. From teasing this relationship apart by examining the environmental quality domains separately, we conclude that the air quality domain primarily explains the observed significant effect modifications.

Previous studies, examining effect modifications of air pollution on temperature-related mortality, were inconclusive–some studies found non-significant effects while others found significant effect modifications [16]. Most of these studies focused on ozone, because it is associated with both mortality and temperature [16]. For example, ozone concentrations are typically higher in the summertime, and a high ozone concentration can lead to difficulty in breathing and aggravation of respiratory diseases [37]. Our study indeed found a positive effect modification between air quality and HI on NAD. This result was consistent with the study of Ren et al. (2006) [14], which found a positive effect modification of ozone on temperature-related cardiovascular mortality. This provides additional evidence that air quality is likely to influence temperature-related mortality. In fact, air quality was observed to have the strongest modification effect among the five environmental quality domains studied. This result suggests that air quality should be taken into consideration in the design of future studies aiming to quantify the health effects of heatwaves.

Furthermore, most previous studies on the interactions between temperature and air pollution on mortality only focused on a single or a few air pollutants (such as ozone or particular matter) [16]. Effect modifications by these air pollutants on the temperature-mortality association varied. The present study investigated interactions between the cumulative air quality and heatwaves. The air EQI used in this study included 87 air pollutants [17]. Thus, it can provide a more comprehensive measurement of the air quality that specific populations are exposed to.

Previous studies have found that populations with low socioeconomic statuses are more susceptible to temperature-related harmful health effects, particularly populations without air conditioning [6,11]. In our study, the trend for associations across tertiles of sociodemographic EQI were flat for all three heatwave indices. The sociodemographic EQI mainly included variables concerning housing, poverty, education, employment, and crimes. These variables can be highly heterogeneous across a county. Thus, the insignificant interactions between sociodemographic EQIs and HI may be the result of exposure mismatching–the county-level sociodemographic EQI may not accurately represent the sociodemographic environment that each individual experiences. In addition, individual-level sociodemographic variation is accounted for within the case-crossover design, therefore, additional differences based on area-level sociodemographic characteristics may be minimal in this population. Future studies using environmental exposure on a more detailed spatial scale (such as census tracts or zip code levels) may address this question.

The definition of heatwaves can vary by space and time [4,24,38]. In this study, we focused our analysis on three indices previously shown to be associated with non-accidental death (NAD) and preterm birth in Alabama [4], to explore effect modifications by environmental quality characteristics. As other researchers have noted [38], defining heatwave indices locally, based on adverse health outcomes, is important for developing public health interventions to mitigate the effects of extreme heat events on health outcomes.

One potential limitation of this study is that the time periods for EQI (2000–2005) did not fully cover the time range for mortality and HI data (1997–2010). However, many of the environmental variables included in the EQI remain relatively stable across years. Thus, the EQI may still be representative of the cumulative environmental quality for the full study period. Another limitation is that our analyses only included metropolitan counties in Alabama. Given the potential heterogeneity in environmental exposures across regions and across the rural-urban gradient, the results of this study may not be generalizable to a wider area.

A key strength of this study was the use of the EQI to represent the cumulative environmental quality. Although populations are usually exposed to a variety of environmental factors at the same time, no previous studies have explored the effect modifications of multiple environmental factors on the heatwave-mortality relationship. The EQI used in this study provides a tool to approach unaddressed research questions in this area. Compared to previous studies using single environmental exposures, the use of EQI is more likely to capture the health effects resulting from the overall burden of environmental exposures [18,19].

## 5. Conclusions

Our results suggest that associations between heatwaves and NAD differ in counties with different cumulative environmental qualities. Larger positive associations between heatwaves and NAD were observed in counties with worse overall cumulative environmental qualities. Among the five domains of environmental quality, an effect modification was observed for air quality. The effect modification of environmental quality on the association between heatwaves and NAD has important implications for operational warning systems and for studies designed to quantify health impacts of heatwaves. The study also demonstrated that EQI and its five domain indices are a useful tool to represent the cumulative environmental quality and different aspects of the environment at the county-level.

## Figures and Tables

**Table 1:** Summary of data on the Environmental Quality Index (EQI), Heatwave Indices (HI) and non-accidental deaths in Alabama metropolitan counties 1997–2010.

EQI Tertiles	Number of Counties	Number of Deaths	Number of Deaths during HI01	Number of Deaths during HI02	Number of Deaths during HI07
Overall 1	10	13,805	155	651	243
Overall 2	9	46,704	520	2015	812
Overall 3	9	79,722	1210	4306	1697
Air 1	10	14,931	162	660	243
Air 2	9	29,501	401	1567	565
Air 3	9	95,799	1322	4745	1944
Water 1	10	19,460	264	959	357
Water 2	9	28,563	409	1609	623
Water 3	9	92,208	1212	4404	1772
Land 1	10	39,293	413	1602	653
Land 2	9	33,677	421	1591	627
Land 3	9	67,261	1051	3779	1472
Built 1	10	12,582	145	558	218
Built 2	9	44,342	491	1968	791
Built 3	9	83,307	1249	4446	1743
Sociodemographic 1	10	36,076	518	1862	746
Sociodemographic 2	9	77,561	1121	4132	1566
Sociodemographic 3	9	26,594	246	978	440

**Table 2:** Percent change in odds ratio (mean and (95% CI)), comparing heatwave days and non-heatwave days by tertiles of overall EQI and EQI domain indices, in metropolitan counties in Alabama. A higher EQI tertile represents a worse environmental quality.

	Percent Change in Odds Ratio (95% CI)		
EQI Tertiles	HI01	HI02	HI07
Overall 1	−10.29 (−26.60, 9.64)	−5.84 (−14.92, 4.19)	−16.25 (−29.55, −0.45)
Overall 2	−7.29 (−16.95, 3.49)	0.47 (−5.04, 6.29)	−2.26 (−10.92, 7.24)
Overall 3	13.22 (4.92, 22.18)	8.26 (4.00, 12.70)	15.44 (7.40, 24.07)
Air 1	−12.99 (−28.54, 5.95)	−6.93 (−15.77, 2.83)	−16.25 (−29.37, −0.69)
Air 2	−6.59 (−17.87, 6.22)	3.87 (−2.75, 10.95)	−4.26 (−14.86, 7.65)
Air 3	10.89 (3.22, 19.12)	6.34 (2.41, 10.43)	12.75 (5.66, 20.32)
Water 1	16.07 (−1.25, 36.43)	9.15 (0.28, 18.80)	3.05 (−10.78, 19.03)
Water 2	−4.75 (−16.21, 8.28)	0.48 (−5.90, 7.30)	−3.71 (−14.22, 8.10)
Water 3	5.33 (−2.19, 13.43)	4.84 (0.84, 8.99)	9.59 (2.48, 17.19)
Land 1	−3.68 (−14.66, 8.71)	0.48 (−5.62, 6.98)	−0.89 (−10.40, 9.62)
Land 2	5.17 (−7.36, 19.40)	5.75 (−0.94, 12.89)	6.54 (−4.76, 19.19)
Land 3	8.03 (−0.44, 17.21)	5.72 (1.28, 10.35)	9.33 (1.14, 18.19)
Built 1	−6.11 (−23.95, 15.93)	−2.76 (−12.83, 8.47)	−4.26 (−20.20, 14.88)
Built 2	−5.45 (−15.35, 5.60)	1.28 (−4.39, 7.28)	−0.39 (−9.46, 9.59)
Built 3	10.97 (2.91, 19.67)	6.89 (2.77, 11.17)	10.69 (3.19, 18.73)
Sociodemographic 1	−1.83 (−12.38, 9.99)	2.23 (−3.82, 8.66)	6.77 (−4.09, 18.86)
Sociodemographic 2	8.81 (0.51, 17.80)	5.25 (1.06, 9.61)	5.24 (−2.16, 13.20)
Sociodemographic 3	0.38 (−13.57, 16.58)	4.91 (−3.13, 13.62)	5.31 (−6.61, 18.76)

EQI: Environmental Quality Index; HI: Heatwave Index.

## Supplementary Materials

The following are available online at www.mdpi.com/1660-4601/14/10/1143/s1, Figure S1: Tertiles for air EQI in metropolitan counties in Alabama. Non-metropolitan counties are white. Tertile 1: counties with the best air qualities in metropolitan areas; tertile 2: counties with median level air qualities; tertile 3: counties with the worst air qualities, Figure S2: Tertiles for water EQI in metropolitan counties in Alabama. Non-metropolitan counties are white. Tertile 1: counties with the best water qualities in metropolitan areas; tertile 2: counties with median level water qualities; tertile 3: counties with the worst water qualities, Figure S3: Tertiles for land EQI in metropolitan counties in Alabama. Non-metropolitan counties are white. Tertile 1: counties with the best land environments in metropolitan areas; tertile 2: counties with median level land environments; tertile 3: counties with the worst land environments, Figure S4: Tertiles for built EQI in metropolitan counties in Alabama. Non-metropolitan counties are white. Tertile 1: counties with the best built environments in metropolitan areas; tertile 2: counties with median level built land environments; tertile 3: counties with the worst built environments, Figure S5: Tertiles for sociodemographic EQI in metropolitan counties in Alabama. Non-metropolitan counties are white. Tertile 1: counties with the best sociodemographic environments in metropolitan areas; tertile 2: counties with median level sociodemographic environments; tertile 3: counties with the worst sociodemographic environments, Table S1: Coefficients for the Interaction term (mean and (95% CI)) comparing the associations between heatwave indices (HIs) and non-accidental death (NAD) in EQI tertile 2 and 3 to EQI tertile 1.
