# Supplementary material for: Effect Modification by Environmental Quality on the Association between Heatwaves and Mortality in Alabama, United States
Source: Int J Environ Res Public Health. 2017 Sep 28;14(10):1143. doi: 10.3390/ijerph14101143 (PMC5664644; doi:10.3390/ijerph14101143)
Supplement: Supplementary file 1 [file ijerph-14-01143-s001.pdf]

Table S1. Coefficients for the Interaction term (mean and (95% CI)) comparing the associations between heatwave indices (HIs) and non-accidental death (NAD) in EQI tertile 2 and 3 to EQI tertile 1.

	Difference in Coefficients (95% CI)		
**EQI Tertiles**	**HI 01**	**HI 02**	**HI 07**
Overall 2	0.03 (−0.20, 0.26)	0.06 (−0.05, 0.18)	0.15 (−0.04, 0.35)
Overall 3	0.23 (0.02, 0.45)	0.14 (0.03, 0.25)	0.32 (0.13, 0.51)
Air 2	0.07 (−0.16, 0.31)	0.11 (−0.01, 0.23)	0.13 (−0.07, 0.34)
Air 3	0.24 (0.03, 0.45)	0.13 (0.03, 0.24)	0.30 (0.11, 0.48)
Water 2	−0.20 (−0.40, 0.01)	−0.08 (−0.19, 0.02)	−0.07 (−0.25, 0.12)
Water 3	−0.10 (−0.27, 0.08)	−0.04 (−0.13, 0.05)	0.06 (−0.10, 0.22)
Land 2	0.09 (−0.09, 0.26)	0.05 (−0.04, 0.14)	0.07 (−0.08, 0.22)
Land 3	0.11 (−0.03, 0.26)	0.05 (−0.03, 0.13)	0.10 (−0.03, 0.23)
Built 2	0.01 (−0.23, 0.24)	0.04 (−0.08, 0.16)	0.04 (−0.17, 0.25)
Built 3	0.17 (−0.06, 0.39)	0.09 (−0.02, 0.21)	0.15 (−0.05, 0.34)
Sociodemographic 2	0.10 (−0.04, 0.24)	0.03 (−0.04, 0.10)	−0.01 (−0.14, 0.12)
Sociodemographic 3	0.02 (−0.17, 0.21)	0.03 (−0.07, 0.13)	−0.01 (−0.17, 0.15)

EQI: Environmental Quality Index.

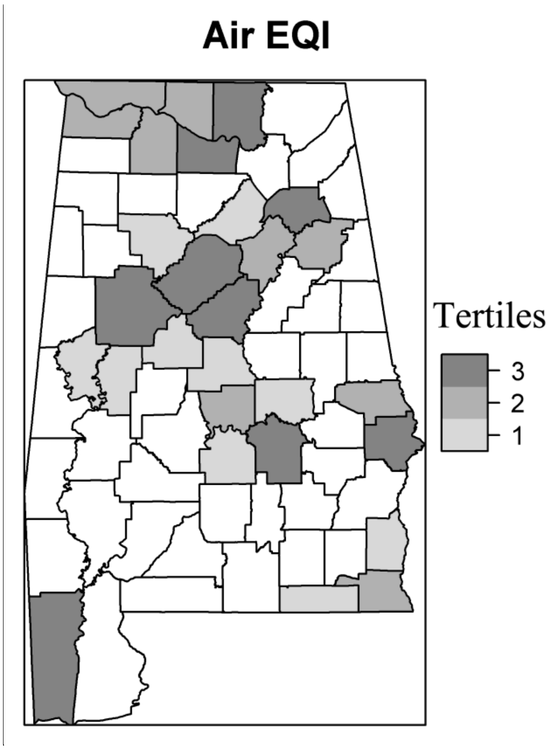

Figure S1. Tertiles for air EQI in metropolitan counties in Alabama. Non-metropolitan counties are white. Tertile 1: counties with the best air qualities in metropolitan areas; tertile 2: counties with median level air qualities; tertile 3: counties with the worst air qualities.

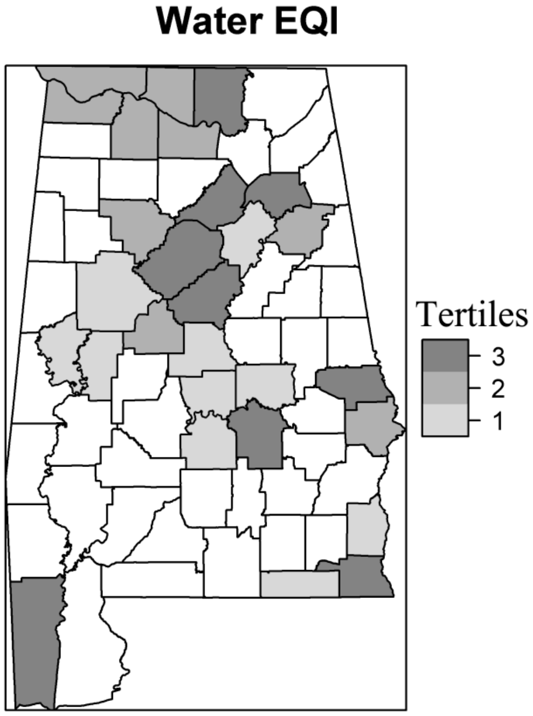

Figure S2. Tertiles for water EQI in metropolitan counties in Alabama. Non-metropolitan counties are white. Tertile 1: counties with the best water qualities in metropolitan areas; tertile 2: counties with median level water qualities; tertile 3: counties with the worst water qualities.

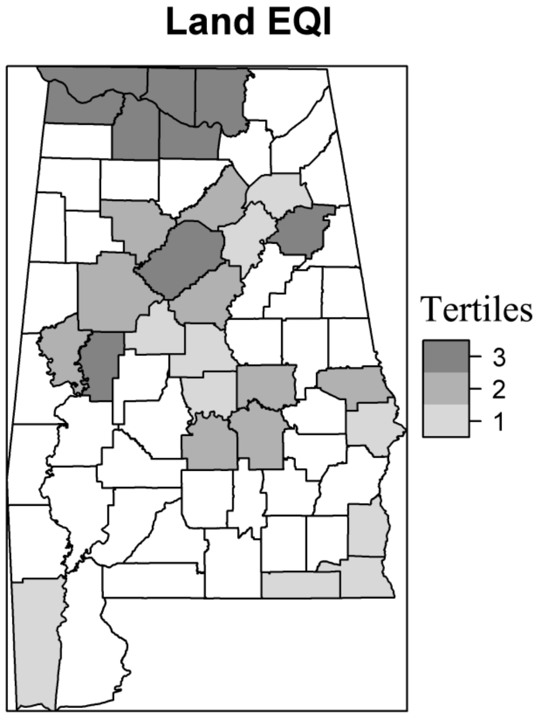

Figure S3. Tertiles for land EQI in metropolitan counties in Alabama. Non-metropolitan counties are white. Tertile 1: counties with the best land environments in metropolitan areas; tertile 2: counties with median level land environments; tertile 3: counties with the worst land environments.

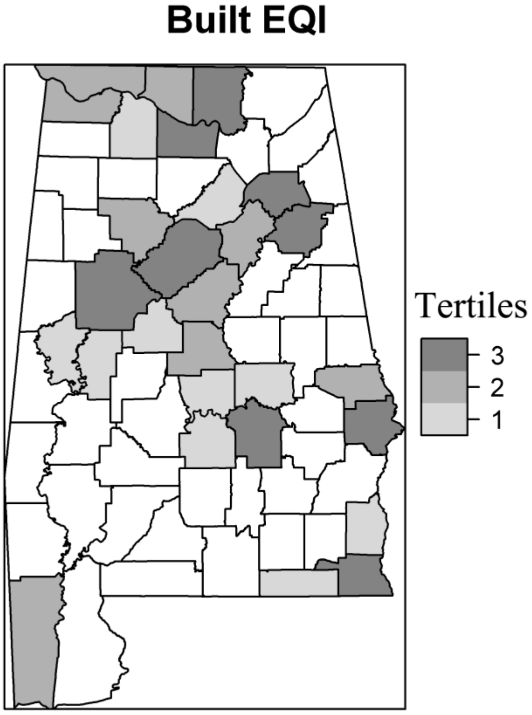

Figure S4. Tertiles for built EQI in metropolitan counties in Alabama. Non-metropolitan counties are white. Tertile 1: counties with the best built environments in metropolitan areas; tertile 2: counties with median level built land environments; tertile 3: counties with the worst built environments.

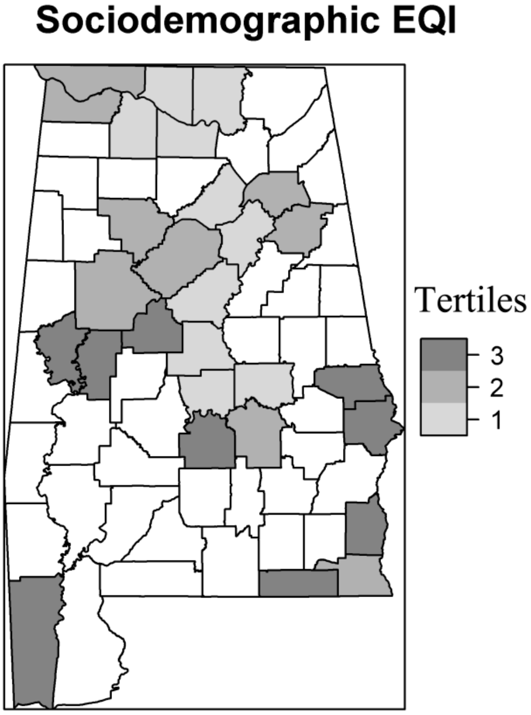

Figure S5. Tertiles for sociodemographic EQI in metropolitan counties in Alabama. Non-metropolitan counties are white. Tertile 1: counties with the best sociodemographic environments in metropolitan areas; tertile 2: counties with median level sociodemographic environments; tertile 3: counties with the worst sociodemographic environments.
